# A cost-effectiveness analysis of fluoride varnish application in preventing Root caries in elderly persons: a Markov simulation study

**DOI:** 10.1186/s12903-024-04226-5

**Published:** 2024-04-22

**Authors:** Long-Wen Zhu, Rui-Xin Wang, Yu Zhang, Jing-Yu Zhan, Hai-Xia Lu, Xi Chen

**Affiliations:** 1 Department of Preventive Dentistry, Shanghai Ninth People’s Hospital, College of Stomatology, National Center for Stomatology, Shanghai Key Laboratory of Stomatology, Shanghai Jiao Tong University School of Medicine, Shanghai Jiao Tong University, National Clinical Research Center for Oral Diseases, Shanghai Research Institute of Stomatology, 639 Zhizaoju Road, Shanghai, China; 2 School of Public Health, Fudan University, Shanghai, China

**Keywords:** Cost-effectiveness analysis, Root caries, Sodium fluoride varnish, Elderly persons

## Abstract

**Background:**

Root caries are prevalent issues that affect dental health, particularly among elderly individuals with exposed root surfaces. Fluoride therapy has shown effectiveness in preventing root caries, but limited studies have addressed its cost-effectiveness in elderly persons population. This study aimed to evaluate the cost-effectiveness of a fluoride treatment program for preventing root caries in elderly persons within the context of Chinese public healthcare.

**Methods:**

A Markov simulation model was adopted for the cost-effectiveness analysis in a hypothetical scenario from a healthcare system perspective. A 60-year-old subject with 23 teeth was simulated for 20 years. A 5% sodium fluoride varnish treatment was compared with no preventive intervention in terms of effectiveness and cost. Tooth years free of root caries were set as the effect. Transition probabilities were estimated from the data of a community-based cohort and published studies, and costs were based on documents published by the government. The incremental cost-effectiveness ratio (ICER) was calculated to evaluate cost-effectiveness. Univariate and probabilistic sensitivity analyses were performed to evaluate the influence of data uncertainty.

**Results:**

Fluoride treatment was more effective (with a difference of 10.20 root caries-free tooth years) but also more costly (with a difference of ¥1636.22). The ICER was ¥160.35 per root caries-free tooth year gained. One-way sensitivity analysis showed that the risk ratio of root caries in the fluoride treatment group influenced the result most. In the probabilistic sensitivity analysis, fluoride treatment was cost-effective in 70.5% of the simulated cases.

**Conclusions:**

Regular 5% sodium fluoride varnish application was cost-effective for preventing root caries in the elderly persons in most scenarios with the consideration of data uncertainty, but to a limited extent. Improved public dental health awareness may reduce the incremental cost and make the intervention more cost-effective. Overall, the study shed light on the economic viability and impact of such preventive interventions, providing a scientific basis for dental care policies and healthcare resource allocation.

## Introduction

Root caries are common dental problems that primarily affect the root surfaces of teeth, especially in elderly individuals who have gingival recession and exposed root surfaces [1]. The prevalence of root caries varies across studies. According to the latest national survey in China, it has been reported that approximately 60% of adults over the age of 65 have experienced root caries [2]. In addition to the higher incidence of exposed root surfaces due to gingival recession, the condition is exaggerated in elderly persons due to factors such as age-related changes in saliva production, diminished oral hygiene practices, and the presence of systematic medical conditions that may impact overall immunity [3]. Root caries can have significant adverse effects on the oral health of elderly individuals. As the tooth roots become demineralized and decayed, individuals may experience symptoms, such as tooth sensitivity, pain, and chewing difficulty. Root caries can eventually lead to pulp inflammation, infection, and potential tooth loss, which significantly affect an individual’s overall well-being, nutrition, and quality of life [4].

Fluoride therapy plays a key role in caries prevention and has been proven to be effective in preventing root caries in elderly persons [5], acting by reducing the solubility of dental tissue, enhancing remineralization, and inhibiting cariogenic bacteria [6]. Fluoride therapy can be delivered through various methods, such as high fluoride concentration toothpaste, mouth rinse, varnish, or gel. All of them are easy to perform and noninvasive, which is favorable for elderly individuals.

When it comes to addressing root caries that have already developed, restoration is the most common treatment for root caries. However, restorations may fail and require retreatment as well as additional costs. Moreover, pulp-affected cases need further endodontic treatment, and crown restoration is suggested afterward, which is very expensive. In severe cases, there is no choice but to extract root caries-affected teeth. Those teeth are prone to fracture during the extraction and may require extra efforts or more experienced dentists to accomplish, which means higher cost and, most unfavorably, the loss of natural teeth. On the other hand, preventive methods, such as fluoride therapy, are more affordable for a single application than treatment for root caries, which seems to be a more cost-effective alternative, but they need to be performed repeatedly, and over time, the cumulative costs may become significant.

Studies on the cost-effectiveness of caries prevention mostly target children, while few specifically target adults and especially elderly persons [7]. Therefore, the present study aimed to evaluate the cost-effectiveness of a fluoride varnish application program in preventing root caries among elderly persons compared with no specific intervention in the context of Chinese public healthcare. The analysis was performed by establishing a Markov simulation model of root caries in a hypothetical scenario. The results would provide a scientific basis for decision makers aiming to optimize healthcare resource allocation.

## Methods

The study was reported according to the Consolidated Health Economic Evaluation Reporting Standards (CHEERS) [8].

### Model and setting

**Fig. 1:**
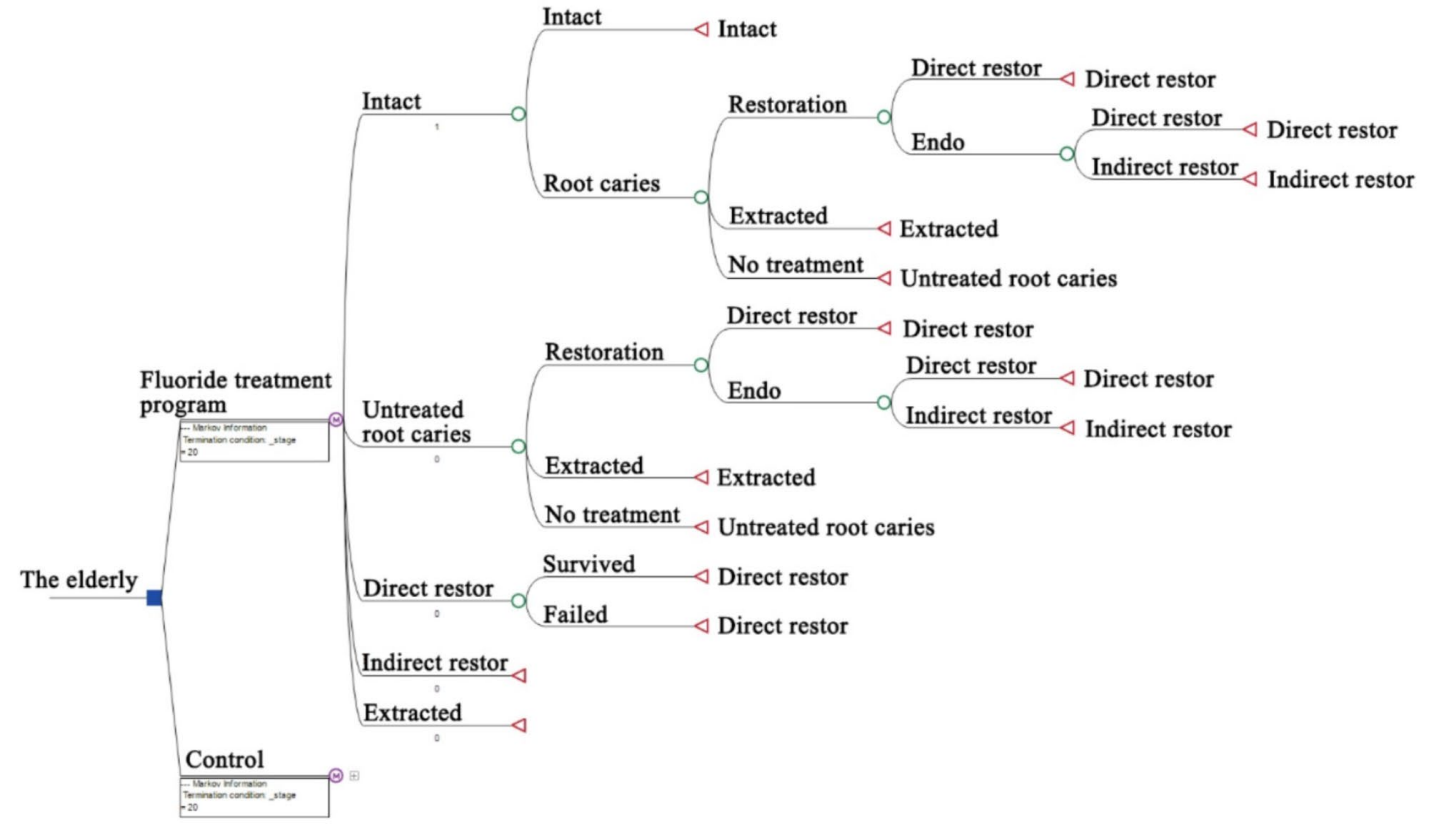
Decision tree of the Markov model. The subsequent structure of the control was identical to fluoride treatment. Subjects in both groups would be at risk of developing new root caries based on corresponding probabilities in each cycle. The decayed teeth could be restored (with or without endodontic treatment), extracted or remain untreated. A proportion of endodontically treated teeth would undergo crown restoration afterward. The decayed teeth that did not receive any treatment would transition to untreated root caries status in the next cycle and faced the same possible transitions as the previous cycle. Teeth in direct restoration status would have a risk of restoration failure in each cycle and require refilling. Crown restoration teeth as well as extracted teeth were regarded as constant. Abbreviation endo, endodontic treatment; restor, restoration

A Markov simulation model was adopted for the cost-effectiveness analysis of a hypothetical scenario. The study was from a healthcare system perspective. The model involved a 60-year-old subject with 23 teeth, which was the average number of remaining teeth among Chinese elders based on a national survey [2]. The time horizon was set at 20 years in the model, which was performed in annual cycles. The analysis was based on tooth levels. A subject would begin with all the teeth intact and would transition among several possible health statuses, including root caries, direct restoration (filling), indirect restoration (crown), or tooth loss, depending on certain probabilities (Fig. 1). We set the tooth years free of root caries as the effect (a subject free of any root caries through the 20 years was considered to have 460 root caries-free tooth years) and the model was based on the assumption of no tooth loss due to other oral conditions, such as periodontitis or dental trauma. The aggregate expenses resulting from root caries, combined with the expense of fluoride application in the possible fluoride treatment program, represented the costs of the associated strategy. We calculated the incremental cost-effectiveness ratio (ICER) to compare the costs and effects of the fluoride treatment program with the situation without intervention. As was adopted by previous studies of cost-effectiveness analysis in caries prevention [9, 10], we set a willingness-to-pay (WTP) threshold based on the monetary cost of a filling (direct restoration) of root caries tooth in Chinese public healthcare system, which was ¥214 [11].

TreeAge Pro 2022 (TreeAge Software, Williamstown, MA, USA) was used for modeling and data analysis.

### Comparators

**Fig. 2:**
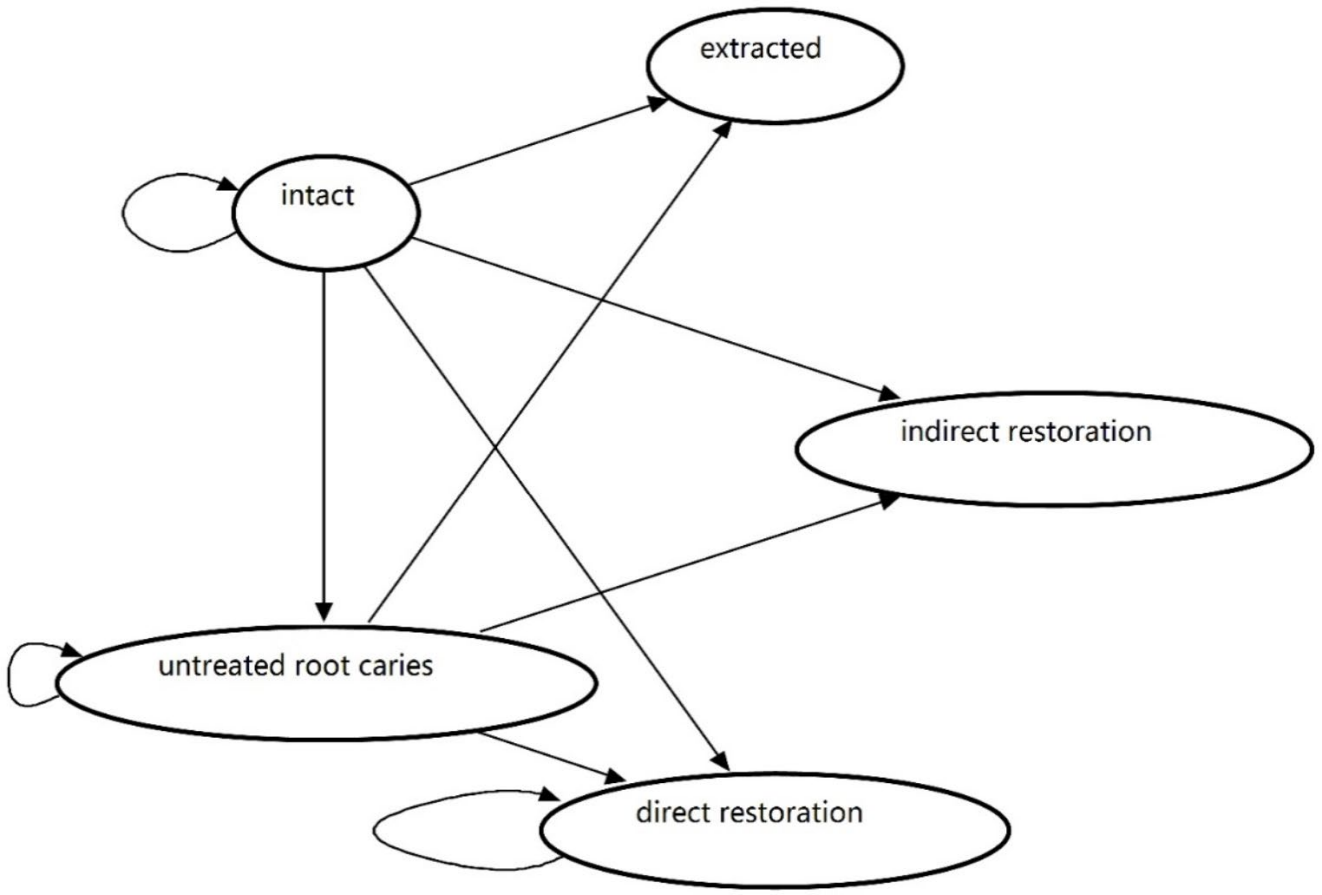
State transition diagram of the Markov model

Subjects involved in the fluoride treatment program were compared with those who did not in a hypothetical scenario. The fluoride treatment program was planned to be a part of an oral health promotion program in development supported by Shanghai Municipal Health Commission, which was aimed at the elderly aged ≥ 60 years. The program was intended to involve oral hygiene instruction and education that promote oral health awareness as well as routine fluoride varnish application for prevention of common oral disease in the elderly. The program is planned to take place in participating medical institutions and provided by associated dental practitioners. Notably, 5% sodium fluoride varnish, proven to be effective in preventing new root caries [12], was applied to all the remaining teeth with exposed surfaces for subjects in the program. The application frequency was once every 3 months in the base case. No specific prevention treatment was performed in the control group. Subjects in both groups would have a risk of developing new root caries based on corresponding probabilities at each cycle. The decayed teeth could be restored (fillings) with or without endodontic treatment, depending on whether pulp or periapical tissue was affected. A proportion of endodontically treated teeth would receive crown restoration afterward. Teeth with severe root caries were extracted. The decayed teeth that did not receive any treatment would transition to untreated root caries status in the next cycle and faced the same possible transitions as the previous cycle. Teeth in direct restoration status would have a risk of restoration failure at each cycle and require refilling. Crown restoration teeth as well as extracted teeth were regarded as constant in the model (Fig. 2).

### Parameters

Transition probabilities were estimated from the data from the Taizhou Imaging Study (TIS), which was an ongoing community-based cohort nested in the Taizhou Longitudinal Study [13], and other published studies (Table 1). Transition probabilities were assumed to be constant in the model. Data that were not collected on an annual basis were converted by the formula p_0_ = 1-(1-p_1_)^1/n^ [14]. Transition probabilities were at the tooth level, and each tooth was considered independent of each other in the model. The TIS recorded detailed oral health condition data of every tooth of every subject, therefore facilitated us to summarize and calculate the average transition probabilities at tooth level. The probability of root caries in the fluoride treatment group was estimated by multiplying the risk ratio of root caries in teeth treated with 5% sodium fluoride varnish by the probability of root caries of teeth in the non-fluoride treatment group.

**Table 1:** Transition probabilities in the base case and sensitivity analysis

Transition probabilities	Base case value	Range of one-way sensitivity analysis^a^	Probabilistic sensitivity analysis		Reference
			α	β	
Intact tooth to root caries	0.007	0.005–0.008	79	11,655	TIS [13]
Root caries to restoration	0.096	0.062–0.130	27	258	TIS
Restoration to endo treatment	0.268	0.157-0.690^b^	146	399	Sun et al. [15] Wang et al. [16]
Crown restoration after endo treatment	0.640	0.349-0.757^b^	281	158	Chu et al. [17] Qiu et al. [18]
Root caries to extraction	0.088	0.055–0.121	25	260	TIS
Direct restoration failure	0.079	0.012–0.146	5	58	Lo et al. [19]
RR_root caries_ in Fluoride treatment group	0.396	0.223–0.702	0.396^c^	0.097^c^	Tan et al. [12]

*Abbreviation* TIS, Taizhou Imaging Study; RR, risk ratio

^a^Range of one-way sensitivity analysis was estimated from the 95% confidence interval of the base case value except where noted

^b^Range of transition probability of restoration to endo treatment and crown restoration was estimated from the minimum and maximum of reference data

^c^β distribution parameters of RR_root caries_ in fluoride treatment group were set from the mean and 10% SD of the base case value

The model was based on the public healthcare system, as it is most accessible to the majority of Chinese people and the most common approach for dental care. The study was from healthcare system perspective, which involved only the monetary costs directly from healthcare service that included both the portion of public payer coverage and the portion of patients’ out-of-pocket cost. Therefore, costs were estimated according to the Summary of Shanghai Medical Institution Medical Service Items and Fees [11] published by the government, which represented the market prices at the time of the study (2023). A certain therapy or treatment is disassembled into steps and items according to the summary. For instance, root canal therapy is disassembled into access preparation, canal preparation, disinfection, obturation, etc., and the total cost depends on the specific techniques of those steps and materials being used. The warm vertical condensation technique costs more than traditional cold lateral condensation. Thus, the cost of a therapy or treatment varies based on factors including the severity of the disease, dentist’s technique, patient preference, etc. The costs of the most common combinations were determined to be the base case value of a certain treatment (Table 2). The registration fee is a constant expense charged each time a patient visits a dentist, and the value is based on the level of the medical institution and the type of clinic. A tertiary medical institution, the general clinic, was considered in the base case for treatment of root caries teeth because it was the most preferred health care service by the Chinese public [20, 21]. Fluoride varnish treatment did not contain registration fee as it was supposed to be provided in the previously mentioned oral health promotion program that did not involve in the hierarchical medical system.

**Table 2:** Costs in the base case and sensitivity analysis

		Base case value CNY¥ (USD$)	Range of one-way sensitivity analysis	
Item	Item cost CNY¥		Minimum CNY¥ (USD$)	Maximum CNY¥ (USD$)
**5% sodium fluoride varnish**	8	8 (2.04)	6.4 (1.63) ^a^	9.6 (2.45) ^a^
**Direct restoration**				
Registration fee-tertiary general	22	•^b^		
Registration fee-primary general	9		•	
Registration fee-tertiary chief	38			•
Pulp vitality test	3	•	•	•
Mobility test	15	•	•	•
Complex filling-composite resin	120	•		•
Simple filling-amalgam	35		•	
Refinement and finishing	14	•	•	•
Indirect pulp capping	20	•		•
Gingivectomy and ESU	190			•
Periapical radiograph	20	•	•	•
Local anesthesia (Primacaïne Adrenaline)	7.06			•
Total		214 (54.59)	96 (24.49)	427.06 (108.94)
**Extraction**				
Registration fee-tertiary general	22	•		
Registration fee-primary general	9		•	
Registration fee-tertiary chief	38			•
Anterior tooth extraction	25		•	
Complex extraction 1	150	•		
Complex extraction 2	300			•
Local anesthesia (Primacaïne Adrenaline)	7.06	•	•	•
Colloidal silver sponge (for hemostasis)	19			•
Periodontal flap surgery	50			•
Periapical radiograph	20	•	•	
Panoramic radiograph	70			•
Antibiotics (Cefradine)	20.6	•		•
Transtelephonic electrocardiographic monitoring	30			•
Total		219.66 (56.04)	61.06 (15.58)	534.66 (136.39)
**Endodontic treatment**				
Registration fee-tertiary general	22	3		
Registration fee-primary general	9		2	
Registration fee-tertiary chief	38			4
Pulp vitality test	3	•	•	•
Mobility test	15	3	2	4
Periapical radiograph	20	4	3	4
Local anesthesia (Primacaïne Adrenaline)	7.06	•	•	•
Rubber dam	15	3		4
Access preparation	5	•	•	•
Pulpectomy	45	•	•	•
Working length measurement	5	2	1	4
Canal disinfection	3	2 × 3	1 × 2	4 × 4
Root canal preparation	25		1	
Root canal preparation w/dental microscope	150	2		4
NiTi rotary instruments	15	2		4
Root canal obturation	55		1	
Warm vertical condensation technique	290	2		4
Resin-based sealer	50	2	1	
Bioceramic-based sealer	150			4
Complex filling-composite resin	120	•		•
Simple filling- amalgam	35		•	
Refinement and finishing	14	•	•	•
Gingivectomy and ESU	190			•
Total		1468.06 (374.51)	358.06 (91.34)	3164.06 (807.16)
**Crown restoration**				
Registration fee-tertiary general	22	2		
Registration fee-primary general	9		2	
Registration fee-tertiary chief	38			2
Impression-silicone	45	2		2
Impression-alginate	15		2	
Impression-agar	20		•	
PFM crown- Co-Cr	1000		•	
PFM crown- noble alloy	2550	•		
All-ceramic crown	1700			•
computer-aided design	2500			•
Cement-RMGI	20	•	•	
Cement-resin	30			•
Provisional crown	20	•	•	•
Cement-ZOE	10	•	•	•
Post and core restoration	470	•		•
Total		3204 (817.35)	1118 (285.20)	4896 (1248.98)

Costs are in CNY¥ except those in parentheses, which are the equivalent costs in USD$. The costs in US dollars were converted based on purchasing power parity according to the International Monetary Fund. (1.00 USD as 3.92 CNY) [22]

The γ-distribution was used for costs, of which the standard deviation was set the same as the mean value

^a^The range of fluoride varnish treatment cost in one-way sensitivity analysis was set as ± 20% of the base case value

^b^Items selected to be involved in certain cases are marked by a dot. If an item was charged multiple times in the treatment, it was marked by the number instead

The effect was set as the tooth years free of root caries. Each tooth remaining in an “intact” state would count as “1” in effect at each cycle; otherwise, it would count as “0”.

A 5% annual discount rate was applied to both costs and effects based on the suggestion of China Guidelines for Pharmacoeconomic Evaluations [23].

### Sensitivity analysis

We conducted one-way sensitivity analysis and probabilistic sensitivity analysis to evaluate the robustness of the results.

### One-way sensitivity analysis.

In the one-way sensitivity analysis, we evaluated the impact of the change of one specific parameter value within a certain range (Table 1). Transition probabilities were mostly set as the 95% confidence interval of the base value, except where noted. For costs, the minimum and maximum of the range was determined by the lowest and highest possible cost of associated treatment (Table 2). For example, a simple anterior tooth extraction without any extra procedures in a general clinic of a primary medical institution led to the lowest possible cost, while a complex extraction 2 procedure (the tooth would tend to fracture during the extraction) in a chief clinic of a tertiary medical institution, combined with periodontal flap surgery, colloidal silver sponge for hemostasis, post-extraction antibiotics and cardiac monitoring if there is any cardiovascular disease risk for the patient, led to the highest possible cost. The range of fluoride varnish treatment cost was set as ± 20% of the base case value. The discount rate ranged from 0 to 8% based on the suggestion of China Guidelines for Pharmacoeconomic Evaluations [23]. The result is depicted in a Tornado diagram.

### Probabilistic sensitivity analysis.

In the probabilistic sensitivity analysis, we evaluated the result of the model when all the related parameters were changed based on certain distributions. The β-distribution was used for transition probabilities (Table 1). The γ-distribution was used for costs, of which the standard deviation was set the same as the mean value. One thousand Monte Carlo simulations were run with the value of each model input being randomly drawn from the assigned parametric distribution. The results were depicted in an incremental cost-effectiveness scatterplot and cost-effectiveness acceptability curves.

## Results

### Base case

We first aimed to evaluate the cost-effectiveness in the base case. In the base case, the total cost incurred by an individual in the fluoride treatment group was ¥2710.22, compared with ¥1074.00 in the control group. The individual achieved 279.67 root caries-free tooth years in the 20-year duration in the fluoride treatment group and 269.46 tooth years in the control group. The ICER was ¥160.35 per root caries-free tooth year gained (Table 3), which was below the ¥214 WTP threshold.

**Table 3:** Incremental cost-effectiveness ratio in the base case

Group	Cost CNY¥ (USD$)	Incremental cost CNY¥ (USD$)	Effectiveness	Incremental effectiveness	ICER
Control	1074.00 (273.98)		269.46		
Fluoride treatment	2710.22 (691.38)	1636.22 (417.40)	279.67	10.20	160.35 (40.91^a^)

Cost is in CNY¥ except values in parentheses, which are the equivalent costs in USD$. The cost in US dollars was converted based on purchasing power parity according to the International Monetary Fund. (1.00 USD as 3.92 CNY) [22]

*Abbreviation* ICER, incremental cost-effectiveness ratio

^a^Incremental cost-effectiveness ratio when incremental cost was converted to equivalent costs in USD$

### One-way sensitivity analysis

**Fig. 3:**
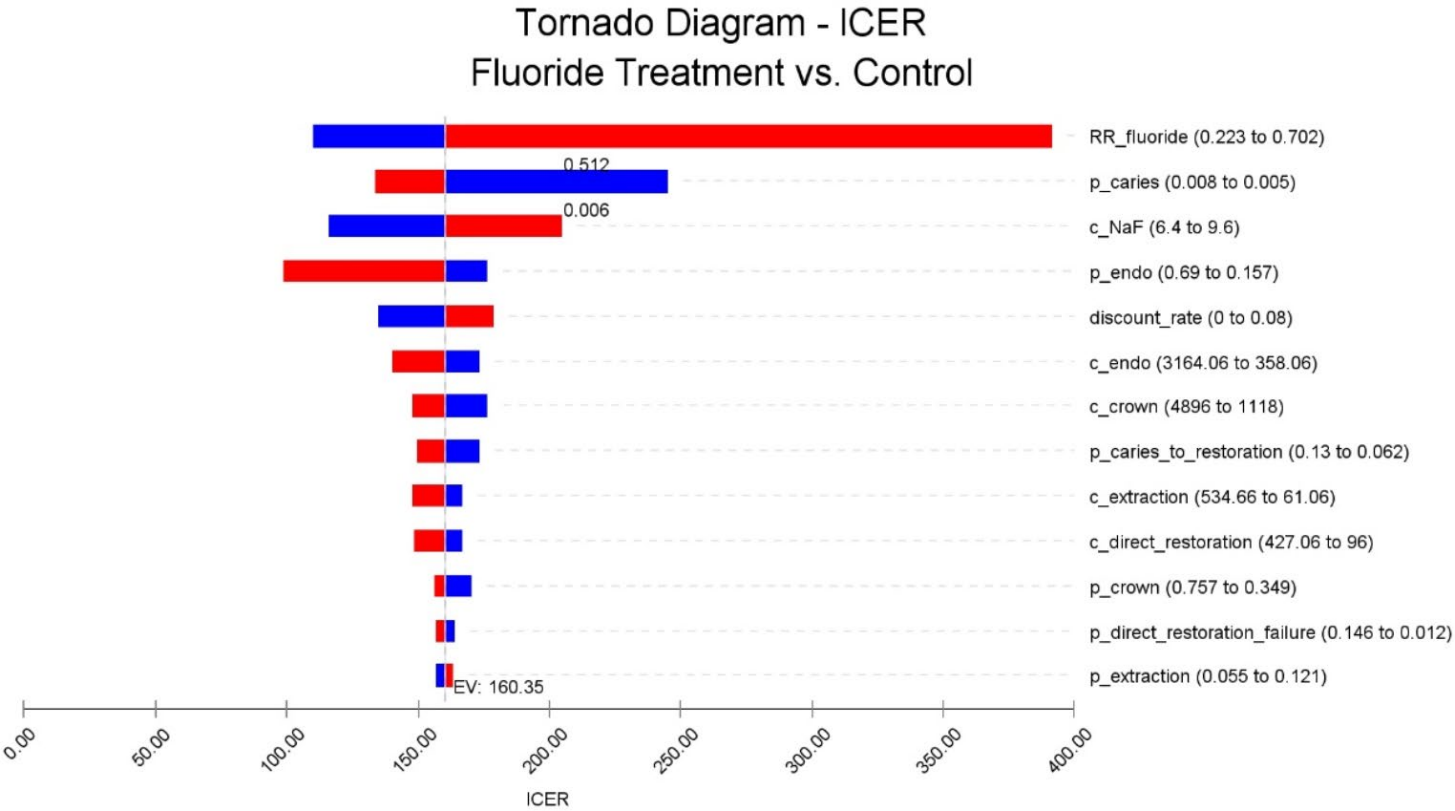
Tornado diagram of the one-way sensitivity analysis. The tornado diagram depicts the range of ICER resulting from the uncertainty of each parameter. It suggests that the risk ratio of root caries in the fluoride treatment group had the greatest impact on the result of the model. The blue part of the bar represents that the ICER increased or decreased from the base case value due to the decrease in the associated parameter, while the red part represents that the change is due to the increase in the parameter. ‘c’ represents cost, and ‘p’ represents transition probability in the names of parameters

One-way sensitivity analysis was performed to evaluate the impact of a change in one specific parameter value. The risk ratio of root caries in the fluoride treatment group had the greatest impact on the result of the model, followed by the transition probability of an intact tooth being root caries and the cost of sodium fluoride varnish. ICER increased from as low as 110.14 to 391.69 as the risk ratio of root caries in the fluoride treatment group increased and from 133.90 to 245.12 as the probability of an intact tooth being root caries decreased. If the risk ratio exceeded 0.512 or the probability of being root caries was below 0.006, ICER would rise north of the ¥214 WTP threshold. For the rest of the situations, ICER would always within the threshold in the one-way sensitivity analysis (Table 4) (Fig. 3). Overall, the result of the one-way sensitivity analysis remained consistent with the base case result in the majority of the situations.

**Table 4:** One-way sensitivity analysis

Parameter	ICER range	Impact
RR_root caries_ in the fluoride treatment group	110.14-391.69	Increase
Probability-intact tooth to root caries	133.90-245.12	Decrease
Cost-NaF varnish	115.80-204.89	Increase
Probability-restoration to endo treatment	98.58-176.59	Decrease
Annual discount rate	134.65-178.81	Increase
Cost-endodontic treatment	140.38-173.41	Decrease
Cost-crown restoration	147.60-176.06	Decrease
Probability-root caries to restoration	149.90-173.66	Decrease
Cost-extraction	147.66-166.73	Decrease
Cost-direct restoration	148.28-167.03	Decrease
Probability-crown restoration after endo treatment	156.33-170.32	Decrease
Probability-direct restoration failure	156.67-164.02	Decrease
Probability-root caries to extraction	156.84-163.27	Increase

All costs are in CNY¥

*Abbreviation* RR, risk ratio

### Probabilistic sensitivity analysis

**Fig. 4:**
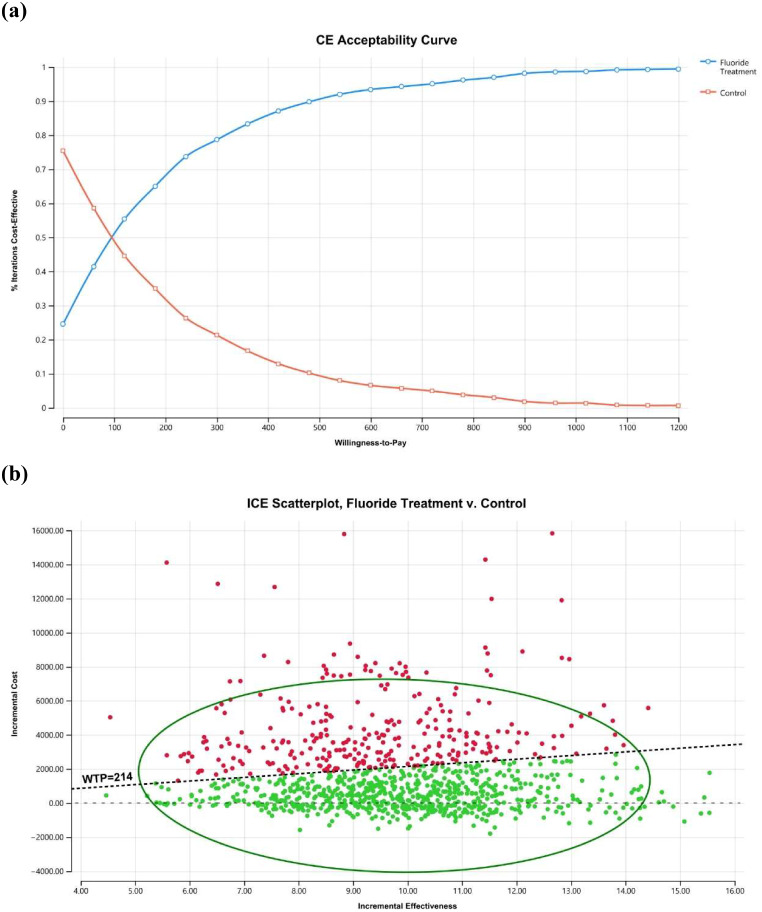
The cost-effectiveness acceptability curves and incremental cost-effectiveness scatterplot results from the probabilistic sensitivity analysis. (**a**) The cost-effectiveness acceptability curves demonstrate the percentage of iterations of the strategy being cost-effective when a certain willingness-to-pay was given. At a willingness-to-pay of ¥214, the fluoride treatment program was a cost-effective strategy compared with no intervention in approximately 70.5% of the simulated cases. If the WTP increased to ¥480, the prevention strategy would be cost-effective in approximately 90% of the cases. (**b**) The incremental cost-effectiveness scatterplot demonstrates the incremental cost and effectiveness of each simulated case as a dot on the plot. Quadrant IV is the dominant quadrant where the incremental effectiveness is positive while the incremental cost is negative. Simulated cases in the dominant quadrant are always cost-effective regardless of WTP. Simulated cases in Quadrant I are positive in incremental effectiveness but positive in incremental cost, and cost-effectiveness was decided by the willingness-to-pay. A total of 24.6% of the simulated cases fell in the dominant quadrant. The remaining cases were all in Quadrant I

We performed probabilistic sensitivity analysis to evaluate the result of the model when all the related parameters were changed based on certain distributions and obtained following results (Table 5). Both the cost-effectiveness acceptability curves and the incremental cost-effectiveness scatterplot indicated that at a WTP of ¥214, the fluoride treatment program was a cost-effective strategy compared with no intervention in approximately 70.5% of the simulated cases (Fig. 4a and b). If the WTP increased to ¥480, the prevention strategy would be cost-effective in approximately 90% of the cases (Fig. 4a). A total of 24.6% of the simulated cases fell in the dominant quadrant, where the fluoride treatment program brought better effectiveness and reduced the total cost in those situations (Fig. 4b). Overall, the result of the probabilistic sensitivity analysis remained consistent with the base case result that fluoride treatment was a cost-effective strategy in most scenarios.

**Table 5:** Incremental cost-effectiveness ratio in the probabilistic sensitivity analysis

Group	Cost (95% CI) CNY¥	Incremental cost (95% CI) CNY¥	Effectiveness (95% CI)	Incremental effectiveness (95% CI)	ICER (95% CI)
Control	1035.02 (997.06, 1072.99)		270.10 (270.00, 270.21)		
Fluoride treatment	2641.25 (2498.77, 2783.74)	1606.23 (1462.94, 1749.51)	279.86 (279.75, 279.98)	9.76 (9.64, 9.88)	173.83 (157.74, 189.93)

*Abbreviation* CI, confidence interval; ICER, incremental cost-effectiveness ratio

## Discussion

The present study focused on evaluating the cost-effectiveness of a fluoride treatment program in preventing root caries among elderly persons, a topic that has been overlooked for a long time. A healthcare system perspective was chosen in the study because it fit the public-private payer mixed system of China healthcare, of which certain healthcare service items are covered by the public healthcare insurance while the rest needs to be paid by the patients or their various commercial healthcare insurances. The results indicated that fluoride treatment, or regular 5% sodium fluoride varnish application more specifically, was a cost-effective strategy with an ICER below the WTP threshold of ¥214 in most scenarios (70.5% of the simulated cases). But the advantage was to a limited extent as the ICER of base case (¥160.35) was barely below the threshold.

It is a common approach to set 1–3 times gross domestic product (GDP) per capita as the ICER threshold to evaluate the cost effectiveness of an intervention [24, 25]. However, since our study did not use quality adjusted life year (QALY) or disability-adjusted life year (DALY) as the health outcome, GDP per capita was probably not a proper threshold in our case. A WTP of ¥214 was set as the threshold of the cost-effectiveness analysis, which represented the cost for a filling of root caries tooth in Chinese public healthcare system (the same as the cost of direct restoration of base case value). The cost of restoration of a root caries tooth not only involved the ¥120 “Complex filling-composite resin” item, but also items like “Refinement and finishing” and other examination before treatment, which were indispensable steps of a restoration treatment and should be added to the total cost (Table 2). However, as there is an absence of appropriately elicited or generally accepted WTP threshold for oral health-specific outcomes [26], it is suggested that decision makers should interpret the results with caution.

As shown in the one-way sensitivity analysis, the risk ratio of root caries in the fluoride treatment group had the greatest impact on the result. Currently, few studies have evaluated the preventive effect of fluoride varnish on root caries [12, 27]. More similar clinical research in the future and summarizing of the results may provide more reliable data as the parameter to alleviate uncertainty.

There were a few possible reasons that the intervention was not dominant over the control. One of the most significant reasons was that the transition probabilities of a root caries tooth to restoration or extraction were relatively low, which indicated that most of teeth with root caries would remain untreated into the next cycle. The low rate of treatment limited the expense caused by the disease and further made the preventive intervention fail to achieve a superior economic result. The transition probabilities of treatment accord with the low root caries filling rate (3.4%) [2] in the national oral health survey in China. This is probably because root caries tends to be neglected by patients unless they become serious enough to have symptoms, such as excessive pain or facture at the cervical part, which usually have a poor prognosis. If the public continues to improve dental health awareness in the future, as national surveys have demonstrated [2, 28], more root caries will be treated at an earlier stage, resulting in an increased cost of the treatment over time, but which can make the intervention more cost-effective.

In addition to 5% NaF varnish, 38% silver diamine fluoride (SDF) solution and 1.23% acidulated phosphate fluoride (APF) gel are also fluoride agents that have proven effective in preventing root caries in elderly individuals [5]. Not only is 38% SDF solution a preferable option for root caries prevention, but it is also considered able to arrest root caries [29, 30] However, no 38% SDF solution product was approved for use in mainland China at the time of the study. Moreover, it may stain tooth tissue and cause aesthetic issue [31]. Therefore, we chose 5% NaF varnish as the agent for fluoride treatment. The situation in which root caries being arrested after fluoride treatment was not considered in the model due to the lack of evidence of the root caries-arresting effect of 5% NaF varnish [5, 32], although the possible arresting effect, if it exists, would lead to a better effectiveness and lower cost in the intervention group and a more favorable overall result.

Only one previous study on the cost-effectiveness of root caries prevention was identified [33]. A similar result (€29.15 per root caries-free tooth year with varnish four times yearly) that root caries prevention was more effective but also more costly in most cases was acquired, despite the difference in preventive agents. In comparison with the previous study, ours adopted a relatively more complex and realistic model, which managed to demonstrate more possible outcomes and associated treatments other than direct restorations immediately at the same cycle. Our model involved the possibility that root caries teeth remaining untreated instead of always treated, which would greatly reduce the costs caused by root caries and potentially result a less cost-effective conclusion of the intervention but closer to reality. On the other hand, further treatment options other than direct restorations might increase the costs and lead to a opposite change to the result. Collectively, our model was expected to produce a relatively more accurate estimation and improve the significance of the results.

There were several limitations in the study. First, the sources of transition probabilities did not have similar scenarios or populations. We tried to gather data on older Chinese adults in recent years. However, limited longitudinal or cohort studies on root caries and related sequelae, let alone multiple studies on certain populations, were found; such studies would have helped us estimate the transition probabilities. Thus, some of the data were based on foreign studies of non-Chinese citizens, conducted decades ago, or were compromised in quality. A real world preliminary longitudinal study to provide data for the analysis, such as what was performed by some other cost-effectiveness analysis studies [34, 35], is a possible solution to improve this drawback. Second, although we choose composite resin for direct restoration in base cases because it is a more common choice for dentists in China, the transition probability of restoration failure is from research on resin-modified glass-ionomer. Sensitivity analysis including the cost of direct restoration and possibility of restoration failure was performed to account for it. Third, although our model was relatively closer to real life cases, simplification was still present due to the great complexity of real-world disease progression. For example, filled root caries can still experience secondary caries, pulp infection or extraction, ultimately, and a crowned tooth may fail and need to bond again or be extracted if fractured. Moreover, the model omitted the process of replacing extracted teeth. However, all of these factors would increase the cost incurred due to root caries and make the intervention more cost-effective to some degree. Finally, all costs were estimated based on the public health system because it is the mainstream approach for dental care in China, but patients can still access private providers [36], which results in more freedom in pricing and are difficult to summarize. Sensitivity analysis on all cost parameters was performed to address the problem.

## Conclusions

In conclusion, regular 5% sodium fluoride varnish application was a cost-effective strategy for preventing root caries in the elderly persons in most scenarios (70.5% of the simulated cases). Improved public dental health awareness may make the intervention more cost-effective. A cohort study in a real scenario could provide more realistic data to improve the accuracy of the analysis.

## Data Availability

All data generated or analyzed during this study are included in this published article.

## Abbreviations

ICER: incremental cost-effectiveness ratio
CHEERS: Consolidated Health Economic Evaluation Reporting Standards
TIS: Taizhou Imaging Study
WTP: willingness to pay
GDP: gross domestic product
QALY: quality-adjusted life year
DALY: disability-adjusted life year
SDF: silver diamine fluoride
APF: acidulated phosphate fluoride
RR: risk ratio
SD: standard deviation
ESU: electrosurgical unit
PFM: porcelain-fused-to-metal
RMGI: resin-modified glass ionomer
ZOE: zinc oxide eugenol
